# On the Evaluation of Engagement in Immersive Applications When Users Are on the Autism Spectrum

**DOI:** 10.3390/s23042192

**Published:** 2023-02-15

**Authors:** Laura Tarantino, Margherita Attanasio, Tania Di Mascio, Giovanni De Gasperis, Marco Valenti, Monica Mazza

**Affiliations:** 1 Department of Information Engineering, Computer Science, and Mathematics, University of L’Aquila, Via Vetoio, 67100 L’Aquila, Italy; 2 Department of Biotechnological and Applied Clinical Sciences, University of L’Aquila, Via Vetoio, 67100 L’Aquila, Italy; 3 Regional Reference Center for Autism of the Abruzzo Region, Local Health Unit ASL 1, 67100 L’Aquila, Italy

**Keywords:** autism, ICT-enhanced ASD treatment, augmented reality, virtual reality, VR solution assessment, social impact

## Abstract

New generation wearable devices allow for the development of interactive environments tailored for Virtual Reality (VR)– and Augmented Reality (AR)–based treatment of Autism Spectrum Disorders (ASD). Experts agree on their potential; however, there is lack of consensus on how to perform trials and the need arises for evaluation frameworks, methods, and techniques appropriate for the ASD population. In this paper, we report on a study conducted with high-functioning ASD people in the 21–23 age range, with the objectives of (1) evaluating the engagement of two headsets offering distinct immersive experiences, (2) reasoning on the interpretation of engagement factors in the case of ASD people, and (3) translating results into general guidelines for the development of VR/AR-based ASD treatment. To this aim, we (1) designed two engagement evaluation frameworks based on behavioral observation measures, (2) set up two packages of reference immersive scenarios, (3) defined the association between metrics and scenarios, and (4) administered the scenarios in distinct sessions for the investigated headsets. Results show that the immersive experiences are engaging and that the apparent lack of success of some evaluation factors can become potential advantages within the framework of VR/AR-based ASD treatment design.

## 1. Introduction

Autism Spectrum Disorders (ASDs) are characterized by fixated and repetitive patterns of behaviors, restricted interests, and social/communication deficit [1,2], severely interfering with the processes of building relationships, integrating and participating into community, and functioning occupationally. Considering the estimated prevalence reported by recent studies (e.g., of about 1.5% in developed countries according to [3], 1 in 68 in the USA [4], about 1 in 100 children in the UK [5], about 0.95% for childhood and adolescence in Central Italy [6]), and the heavy demand caused by ASD people on families and on educational, social, and medical services, there is a significant need for research on therapeutical interventions and (novel) treatment strategies that ensure that persons with ASD achieve optimal outcomes and improve their quality of life, with benefits for ASD people, their families, caregivers, and social networks.

Though the causes of autism remain largely unknown, there is consolidated evidenc on the involvement of genetic, neurodevelopmental, and environmental factors. In particular, deficit in social cognition and its components such as Theory of Mind (ToM, i.e., the ability to understand another’s thoughts, beliefs, and other internal states) is specifically reported in persons with autism [7,8,9]. Given the significant influence that the social cognition model has in the research and practice of ASD, it would be crucial to develop evidence-based intervention strategies based on social cognition abilities [9,10].

### 1.1. ICT-Based ASD Treatment

Notwithstanding the difficulty in predicting which particular intervention approach works best with which ASD individual [2], starting from Colby’s theoretical premises [11] and following the seminal study of Strickland and colleagues [12], in the last two decades literature surveys [13,14,15,16,17,18,19,20,21,22] have witnessed an ever-growing number of proposals for approaches falling in the “Technology-based Treatment” category, as denoted in 2009 by the National Autism Center [23]. The well-established literature regarding the efficacy of the visual modality for ASD people [24,25,26,27], with a recognized preference for visual stimuli transmitted through electronic screen media [25] (such as television screens, computer monitors, tablets, smartphones) and a natural propensity for video and video games [28,29,30,31]), has made Information and Communication Technology (ICT) a first-class option for the investigation of assistive technologies, cognitive rehabilitation tools, and education tools. It must be observed that such research lines did not develop without concerns, at least initially, with fear that the recourse to technology as a treatment tool might exacerbate the isolation of ASD people with difficulties in social relationships [32]. Anyhow, such concerns have been overtaken by evidence on the success of ICT for improving social interaction when used correctly [33]: ICT-based tools are generally accepted and enjoyed by ASD people since interaction with computers does not pose sever expectations and judgement issues and allows the discovery of conventions in safe and predictable environments, supports the imagination of people, contexts, and behaviors necessary for role-play, and offers possibility of modifiable multisensory stimulation, replicability, and the definition of individualized interventions (see, e.g., [34,35,36]).

The continuous advances in computer graphics and input devices (e.g., interactive gloves and eye-trackers), as well as the ample variety of (affordable) devices offering “synthetic experiences” at various levels of immersion, visual and interactive fidelity, and refresh rates have been fostering the investigation on the use of Virtual Reality (VR), Mixed Reality (MR), and Augmented Reality (AR) tools in a variety of ASD applications, such as neurocognitive assessment, psychotherapy, rehabilitation, prevention and treatment of eating disorder, pain management, social skills training, vocational readiness training, and communication training (as surveyed, e.g., in [37]). Computer-generated representations of environments with realistic appearance seem to have a great potential for teaching social understanding, due, among others, to their capability of replicating social situations that may not be feasible in therapeutic settings with space limitations and resource deficits, promoting role-play within such synthetic scenarios (as in [36,38]).

Blascovich [39] defines a Virtual Environment (VE) as a synthetic sensory information that leads to perceptions of environments and their contents as if they were not synthetic, while an Immersive Virtual Environment (IVE) is defined as one that perceptually surrounds an individual such that “immersion in such an environment is characterized as a psychological state in which the individual perceives himself or herself to be enveloped by, included in, and interacting with an environment that provides a continuous stream of stimuli”, as suggested also by [40]. Furthermore, VEs and IVEs can support the interaction of multiple users, favoring group social skills interventions [41]. Parsons singles out two main directions in the exploitation of authenticity of VEs and IVEs: (1) for providing authentic and well-controlled contexts where operators can monitor and assess social responding; and (2) for learning and intervention, as a bridge to the real world for rehearsing specific interactions that can be difficult to practice in other ways [42]. For the latter, of interest for the present study, a significant variety of proposals and scenarios can be found in the literature [43,44,45,46,47,48,49,50,51,52,53,54,55], based on the assumption that interaction with the VE increases the probability that the ASD person transfers learned skills into everyday life, though it has to be said that the evidence base is still limited and small-scale, because of the lack of longitudinal studies supporting such a hypothesis (e.g., [56,57]).

With reference to the consolidated virtuality-continuum proposed by Milgram [58] and depending on the characteristics of the utilized device, existing technology-based ASD treatment proposals can be classified as either based on *augmented-reality* (when “see-through” displays augment the world surrounding the observer with simulated cues) or based on *virtual-reality* (when the virtual environment consists solely of virtual objects), which in turn can be either monitor-based or (at some degree) immersive (thanks to Head-Mounted Displays (HMD) or CAVE environments). It must be said that most proposals are conventional monitor-based applications: the survey in [46] singles out only six empirical studies based on the use HMD technology with autistic population for learning, assessment, and intervention [12,59,60,61,62,63]. Some studies on IVEs are based on CAVE or semi-CAVE environments, where animated images are projected onto the walls and the ceilings of a screened space [64,65]. The study in [66] is based on Google Cardboard, a low-cost device based on smartphones creating the illusion of 3D depth and immersion through the stereoscopic effect generated by the biconvex lenses on the VR viewer and the human vision system. The significant limitation of such CAVE-based and Google Cardboard-based applications derives from the fact that users are spectators unable to interact with the projected objects. Actually, it has to be said that, because of safety reasons, for children under the age of 13 CAVE-based IVEs and HMDs based on stereoscopic effects are the only viable immersive solutions, which—combined with the fact that a great part of the studies is focused on children—may be among the reasons for the scarcity of studies on really immersive HMDs.

Furthermore, notwithstanding the convincing rationale behind the adoption of VR-based technologies in the treatment of ASD and a diffuse optimism, there is still some skepticism about the real effectiveness of VR-based and AR-based interventions in individuals with ASD, and scholars underline the lack of robust studies with strong methodologies and the lack of proof for generalization [17], the little evidence supporting the efficacy of VR technology [18], and the need of more research within educational and clinical settings to ensure robust recommendations can be made on the implementation, use and sustainability of the VR approach [46]. On the other hand, given the availability of a growing number of affordable and accessible VR devices, the assessment of their effectiveness in ASD treatment would grant a pathway for improving the quality of life of ASD persons and their network [37]. Methods and techniques for assessing the efficacy of technology-based treatment and the overall familial and societal impact of their adoption on a large scale are, hence, definitely needed.

### 1.2. Evaluation of VR-Based ASD Treatment

As observed, e.g., in [37], there is lack of consensus on how to perform trials, and there is the need of establishing the psychometric properties of VR assessment and interventions. A first limitation of existing proposals of VR-based and AR-based tools for ASD treatment is the fact that in most cases the evaluation is based on self-reported measures reflecting the perception that a person has about their performance of activities (e.g., the ITC-SoPI questionnaire [67] as in [63] and [65]). Besides the fact that even in the case of Typical Development (TD), people’s responses in self-reports may be over or under estimation of actual abilities [68]; in the ASD case, self-reports are definitely not recommended because of the difficulty that ASD people may have in reflecting and reporting on their own behavior and emotions [69,70].

Furthermore, concepts and criteria used in ASD studies are generally inherited from the evaluation of VEs in general studies on virtuality, making it appropriate to reason on the adequacy of assessment metrics conceived for TD people (as the aforementioned ITC-SoPI questionnaire) in the case of ASD people.

For TD people, the “sense of presence” within a VE (i.e., the sense of being caught up in the representations of virtual worlds [71]) is recognized to be a key factor affecting the way in which VEs are experienced and the interaction taking place within them [42,53,72]. Studies about the relations between users and IVEs typically base the measurement of the efficacy of a VR solution on its capability of replicating the real world, human behavior in real life, and typical human–human interaction. According to Ellis [73], IVEs must give the illusion of displacement to a different location and users have to perceive a high sense of presence. In a seminal study by Slater and Wilbur [74], the immersive capability of an IVE is related to the degree to which it is inclusive, surrounding, extensive, and vivid, and matching, where *the higher the better*. Furthermore, they maintain that the greater the degree of presence, the greater the chance that participants will behave in the IVE, similarly to what they would do in similar circumstances in everyday life. Immersivity and sense of presence are actually considered primary requirements of synthetic worlds, with the implicit assumption of a positive relationship between the user and the real world.

When moving from the TD population to the ASD population, characterized by a profoundly different perception of the reality, it is legitimate to wonder whether and to which extent the same considerations about the sense of presence still hold. In particular, we argue that it may be the case that “higher” is not necessarily always “better” (or just “possible”) when the evaluation of the IVE is within the framework of a therapeutic and/or prosthetic solution for ASD people with core deficits in social communication and interaction, experiencing difficulties in their relationships with the real world or with others. Let us consider, for example, studies on the concept of presence as a performance goal in TD people: Nowak and Biocca reason about various forms of presence, such as the measure of the extent to which the VE is able to “provide access to another mind” (social presence) or the perception of the “psychological connection to and with another person” (copresence) [75]; Riva and colleagues propose a presence model where social presence “allows the Self to identify and interact with Others by understanding their intentions” through the three subprocesses of “other’s presence”, “interactive presence”, and “shared presence”, respectively related to the ability of recognizing “motor intentions in other individuals”, “motor and proximal intentions in other individuals”, and “motor, proximal and distal intentions in other individuals” [72]. It is easy to see that these psychological and cognitive processes require the ability to correctly perceive the self and to understand another’s thoughts and internal states, which is exactly what ASD people lack at different levels of severity. Therefore, we maintain that the achievement of such capabilities can be rather considered among the ultimate goals of an ASD rehabilitation treatment in the long run, than expected to be met when evaluating, e.g., the engagement of an IVE by an ASD person with deficits in social cognition and difficulty in understanding another’s thoughts and belief, which compromise the process of building relationships and integrating into community [76].

### 1.3. Our Study on Engagement Evaluation

All these considerations make the evaluation of VR/AR-based ASD treatment still an open problem with a variety of issues to be addressed.

With reference to the necessity of establishing which psychometric properties have to be considered in the assessment of interventions, we observe that it is recognized that the extent to which someone is persuaded by their experiences in IVEs depends on the degree of involvement or *engagement* that they feel with the content [15,37,77] (e.g., studies about ASD and VEs using physiological markers of engagement, such as pupil dilation and blink rate show that performances of participants improve as engagement increases [35]). Therefore, with the aim of providing a contribution to the open problem, our paper discusses results of a study aimed at evaluating the *engagement capability* and the potential *appropriateness* of two HMDs (Oculus Rift and Microsoft HoloLens, differing in their surrounding extent) with respect to VR-based ASD treatment, to answer the basic research question:

RQ0: *can the immersive experience of an ASD individual with the two selected HMDs be considered engaging (and hence promote the HMDs as potentially appropriate for treatment support)*?

Differently from other existing studies, we conducted the evaluation on the basis of *behavioral observation measures* rather than on self-report characterized by questionable reliability. In particular, in order to reason also on the appropriateness of metrics in the case of ASD people (as discussed in Section 1.2), we defined two distinct sets of engagement metrics: one includes factors typically associated to the evaluation of IVEs independently of the autism condition (realism of the IVE, suspension of disbelief, body participation, exploration, and action), for which it is known that “the higher the better” in the case of TD people, and the other includes factors that can be used to evaluate the engagement in generic situations (facial expression, level of attention, emotional participation, verbal reaction). Possible discrepancies between the results achieved by the two sets of metrics would allow us to provide an answer to the following research question (refining RQ0):

RQ1: *can the immersive experience of an ASD individual be considered actually engaging (and hence promote the HMD as potentially appropriate for treatment support) even when customary IVE-specific evaluation metrics seem to suggest a not optimal level of engagement*?

It may also be the case that, analyzing the lack of success of some “typical” IVE-specific engagement evaluation metrics, we might discover some ASD-specific trait of the connection between the individual and the IVE that can be possibly exploited for the design of an effective VR/AR-based ASD treatment. This leads to a third research question that we aim to address:

RQ2: *can the lack of success in any IVE-specific evaluation metrics turn out to be potentially useful within the framework of IVE-based ASD treatment design*?

The study was conducted with a group of five ASD young people, all high-functioning, aged 21–23. We specifically chose a homogeneous users’ sample representative of a target population potentially highly open to the utilization of state-of-the-art technology and with good chances of integration in the social life, but definitely under-considered in the literature on VR-based ASD treatment (for example, out of the sixteen studies surveyed in [42] only one deals with young adults in the age range 18–26 [34], and, with respect to the users, collectively evaluated by the twenty-nine studies recently surveyed in [52], only 3% are of age 20, while none of these studies reported on ASD people older than 20).

With respect to the selected users’ population, the results achieved allow us to provide positive answers to both the research questions. Furthermore, reasoning about the interpretation of the results in the two evaluation frameworks allows us to outline the nature of VR-based, ASD-oriented applications based on the two HMDs and to suggest possible different roles in ASD treatment, specifically as bridges to the real world, for learning and intervention (with reference to the possible directions of exploitation underlined by Parsons [42]).

The remainder of this paper is organized as follows: in Section 2, after illustrating features and constraints of the equipment used in the experiment and characterizing the participants, we define the evaluation frameworks utilized for measuring acceptability, general usability, and engagement of the selected HMDs and describe the immersive scenarios and the activities administered to experiment participants. Then, while in Section 3 we report gathered data, in Section 4 we discuss these data in relation to ASD treatment made possible by the studied devices and primarily centered on *communication*, *social interaction*, and *autonomy*. Finally, in Section 5, conclusions are drawn and applications of the results in new ongoing projects are outlined.

## 2. Materials and Methods

The study here reported was conducted within the framework of an experience of participatory design carried out at TetaLab (Technology-Enhanced Treatment for Autism Lab), a multidisciplinary laboratory of the University of L’Aquila cooperating with the Regional Reference Center for Autism of the Abruzzo Region. The laboratory was founded by scientists from the Department of Information Engineering, Computer Science and Mathematics and from the Department of Biotechnological and Applied Clinical Sciences, with the aim of conceiving and validating ICT-based ASD treatments specifically centered on *communication*, *social interaction*, and *autonomy*. When the study took place, the TetaLab team included three computer scientists, four psychologists, and one medical doctor; the participatory design activity involved also nine ASD young persons in the age range 15–28; families took part as secondary stakeholders.

The study included two evaluation sessions: the first one took place in Milan at the Microsoft Lab and was focused on augmented reality, while the second one took place in L’Aquila at TetaLab and was focused on immersive virtual reality.

### 2.1. Materials

Coherently with such general goal of the study, we selected two diffused off-the-shelf HMDs offering different interactive experiences with mixed and virtual environments:

- The HoloLens translucent visor, adding a layer of synthetic reality to the natural field of vision that becomes enriched by virtual elements overlaid on top of it. In our evaluation, study participants experimented the Hololens Commercial Suite, which includes the Development Edition hardware as well as enterprise features for added security and device management.
- The Oculus Rift headset, offering a 100% immersion in a virtual world generated by the computer inside the device while the field of view of the real world is cut out. In our experiment, the Oculus Rift headset was connected to a VR-ready laptop (Asus GL 502 V) with Intel Core I-7 7700 HQ, 2.80 GHz clock, 16 GB RAM running the Windows-10 OS and a NVIDIA GTX1070 high performance GPU with 8 GB of dedicated high speed GDDR5 RAM, with 1920 graphic processing cores.

For both devices, official safety warnings [78,79] recommend the utilization by adults and children older than 13, because of vision characteristics (one may observe that this safety constraint translates into a first basic constraint for the typology of conceivable technology-enhanced treatment). Both devices solved cybersickness problems, but adverse effect, such as dizziness, seizures, epileptic seizures or blackouts triggered by light ashes or patterns have been reported in some people (about 1 in 4000), even without history of seizures or epilepsy, more commonly in children and people under the age of 20, and some studies show that cybersickness is a typical cause of withdrawal in HDM studies (e.g., [46,80,81]), both in the typical development population and in the autistic population. The concern about possible adverse effect is clearly greater for the autistic population [82,83,84] and questions related to adverse effects for individuals with ASD when applying VR in general, and HMD-based VR specifically, have been addressed in the literature [46,85,86]. Initial findings provide preliminary evidence supporting safety and usability of HMD-based virtual reality for ASD people [85,86]; however, also given the huge heterogeneity of the autistic population, additional studies are needed in larger samples, larger ranges of VR experiences, and in the context of long-term exposure. Meanwhile, also considering the paucity of research explicitly exploring the potential adverse effects for this vulnerable population [86], precautionary medical supervision may be indicated for the use of HMDs depending on the specific condition of the ASD individual.

### 2.2. Participants

Study participants were recruited within the group of ASD people involved in TetaLab activities. The voluntary nature and the objectives of the study were explained to all TetaLab participants along with logistical information about time, locations, settings and duration of the experiment sessions. Five male ASD individuals aged 21–23, all high functioning and attending either high school or University, chose to take part in the study (a sixth person involved in TetaLab participated in the second evaluation session, but it is not included here to maintain homogeneity both in the comparison among the two HMDs and within the evaluation of the Oculus Rift where the other five participants had previously experienced the first session of evaluation). None of them were under guardianship and all gave informed consent. All participants received a previous diagnosis of ASD (according to DSM-5 [1] and none of them had a history of epilepsy. Participants characterization is shown in Table 1, reporting (1) participants’ demographics, (2) social cognitive characterization, and (3) IQ scores, ranging from 73 to 98 (M = 85.67, SD = 9.89). *Social cognitive characterization* was obtained via the administration of a number of tests: Basic Empathy Scale (BES) [87], Affective Empathy-Basic Empathy [88] AE-BES, Eyes Task (a revised version of the “Reading the Mind in the Eyes Test” [89], and Advanced Theory of Mind (ToM) Task, an Italian adaptation of Blair and Cipolotti cognitive task [90] proposed in the literature by Happè [91]). *IQ scores* were determined according to the Wechsler Adult Intelligence Scale test (WAIS-IV [92]), based on four major components of intelligence (Verbal Comprehension Index (VCI), Perceptual Reasoning Index (PRI), Working Memory Index (WMI), and Processing Speed Index (PSI)).

### 2.3. Measured Factors

Though the investigation was primarily focused on engagement, the overall evaluation framework conceived for the study was more general and included a variety of factors that can be classified in the three macro areas of *acceptability*, *usability*, and *engagement*. Acceptability and usability were measured in order to check whether basic necessary preconditions were satisfied by the selected HMDs, while engagement was measured with the multifold objective of:

1. Evaluating the extent of the connection that study participants developed with the proposed synthetic worlds,
2. Analyzing whether considerations and engagement measures generally valid for TD people could be still considered valid for ASD people, and
3. Translating the achieved results into guidelines in terms of development of technology-enhanced ASD treatment.

The three objectives broadly correspond to the three research questions.

#### 2.3.1. Measured Acceptability and Usability Factors

*Acceptability* was investigated in terms of participants’ willingness to use the evaluated headsets and of a number of factors related to possible unpleasant physiological effects or discomfort (motion-sickness, double vision, digital eye strain) selected based on consolidated literature on IVR and ASD (see, e.g., [63,66,93]) and measured as Boolean values.

*Usability* was investigated according to a variety of aspects related to *autonomy in managing the devices* (measured with respect to support requested to operators during performances and performances in mounting the devices), *comprehension of virtual environment features* (measured with respect to interactive and non-interactive virtual elements, menu navigation elements, menu structure elements) and *interaction ability* (measured with respect to the use of game pad, remote control, gestures). These aspects were measured using a low-medium-high scale.

#### 2.3.2. Measured Engagement Factors

As discussed in the Introduction, engagement was investigated according to two distinct sets of metrics, because our goal was not only to measure the involvement of participants with the experienced IVEs but also to reason on the interpretation of metrics in the case of ASD people. To this end, in ENGAGEMENT FACTORS I (EF_I) we included factors specifically related to the evaluation of IVEs and hence associated to specific activities within the IVEs experienced during the evaluation sessions, while in ENGAGEMENT FACTORS II (EF_II) we included factors associated with the engagement with generic situations and observed during the experience as a whole (we refer to Table 2 for a summary of the two sets).

**ENGAGEMENT FACTORS I**. Aspects in EF_I have been selected taking into account consolidated general literature on VR (i.e., independent of ASD treatment), as follows:

- *emotional participation in watching photorealistic and non-photorealistic images* can provide useful indications on the necessary realism; degree of photorealism is customarily attributed a meaningful role for the achievement of a higher sense of presence [58,74,94], under the assumption that the more realistic an IVE is, the more the scene is believable and the greater is the chance of promoting generalization and transfer of skill and understanding from the virtual to the real world [55];
- *suspension of disbelief* is indicated as a desirable feature to facilitate the interaction with the IVE (e.g., [53,95,96,97]), under the assumption that a VE exerts its measurable influence more by eliciting an acceptance of the virtual world rather than by eliciting a true belief of the realism of the VE [96];
- *body participation* is recognized as having statistically significant relations with the level of engagement [98], and is strictly connected with the concept of presence, being in particular a crucial component of the first level of the self (the “proto presence”), i.e., the ability to enact motor intentions by moving the body as discussed in [72], page 21;
- *active exploration* and *action* are related with self-government exploration, suggested as more efficient than passive avatar-guided exploration of VEs [99]. Furthermore, [100] emphasize that presence and agency are directly related within experiences of using VEs such that “presence is a core neuropsychological phenomenon whose goal is to produce a sense of agency and control: I am present in a real or virtual space if I manage to put my intentions into action (enacting them)”.

**ENGAGEMENT FACTORS II**. Aspects in EF_II has been selected taking into account literature generally focused on interaction with ICT-based tools, without specific reference to IVE experiences. As suggested by [101], everyday connotations of engagement refer to involvement, commitment, passion, enthusiasm, absorption, focused effort, zeal, dedication, and energy. The way an individual engages in an activity is a central component of their experience with the activity [102]. Many researchers have focused on the study of engagement factors during learning processes, emphasizing that engagement implies attentional and emotional involvement with the task [103]. Therefore, the aspects of EF_II were selected taking into account the different emotional and cognitive factors that are often considered in the literature to assess the level of engagement during ICT-based learning processes or, more generally, during interaction with ICT-based tools [102,104,105]. In particular:

- *Facial expressions* are considered indicators of the individual’s affective and cognitive state in real-time, as nonverbal cues containing significant information about the level of attention, involvement, and engagement [102,105,106,107,108].
- *Level of attention* is considered a key component of cognitive engagement during a VR experience [109,110]. Indeed, during interaction, engagement is not stable but can fluctuate. Selective attention to a stimulus seems essential for a basic form of involvement; a more protracted, and therefore focused form of attention, is a requirement for engagement and promotes the possibility of an affective engagement [103].
- *Emotional participation* can be interpreted as the positive affective attitude toward the experience that can motivate the individual to engage in and spend time to the activity [111]. It thus includes pleasure, curiosity, enthusiasm, and anticipatory excitement during and after the experience [112].
- *Verbal reaction* can provide important information about the intensity of involvement. In this regard, verbal behavior, the propensity to comment or ask questions [112] about the activity, can be understood as an indicator of active, motivated, and focused participation during and after the experience.

### 2.4. IVE Scenarios

The package of Immersive Virtual Environment scenarios to be administered to the experiment participants was shaped during a preparatory study where available demos of the two headsets and applications developed at the ICT Living Lab of the Department of Information Engineering, Computer Science and Mathematics of the University of L’Aquila were examined and eventually selected based on their appropriateness to analyze the usability and the engagement factors of the evaluation framework. In particular, the Oculus Rift package included two demos available on the Oculus Store (Interaction to Virtual Reality and the Dreamdeck demos), one application developed at the DISIM ICT Living Lab (3D virtual reality model of the Church of Santa Maria Paganica in L’Aquila [113]), while the HoloLens session included one demo available on the HoloLens Commercial Suite (Michelangelos’ David). Furthermore, the package administered during the Oculus session included also one demo available on the Leap Motion Store (the Blocks game), to evaluate the acceptability and usability of the synthetic representation of one’s hands, a function not available among Oculus Rift demos but potentially viable in Oculus-based applications (to each participant, the demo was administered before the Oculus demos in order not to interfere with HMD-based activities).

All selected HoloLens and Oculus Rift scenarios contributed to the evaluation of all acceptability factors, autonomy in managing devices and comprehension of IVE features (usability factors), suspension of disbelief, body participation and action (EF_1 factors), all EF_II factors. As to emotional participation with respect to photorealism, interaction ability, and exploration of the virtual world, distinct scenarios provided different insights according to their characteristics, as summarized in Table 3 and discussed in detail in the following:

- **E1**: the “Introduction to Virtual Reality” demo allows users to browse different scenes via remote control; for example, users can watch the world from the space as if they were an astronaut, watch the far-away lands as if they were physically in these lands, attend a Cirque du Soleil performance as if they were in the center of the performance itself, interact with a giant from a bygone era as if they were face to face with them. These scenarios and associated activities contributed primarily to the evaluation of *emotional participation in photorealistic images* (EF_1 factor), and *interaction ability in using remote control* (usability factor).
- **E2**: the “Dreamdeck demos” offer a mix of photorealistic and not photorealistic scenarios in which users can, for example, talk with an alien face to face, meet forest animals, watch a city of the future standing on one on its high terrace, moving within a strange museum awaiting the T. Rex coming against them. These scenarios and associated activities contributed primarily to the evaluation of *emotional participation in photorealistic images* and *emotional participation in not photorealistic image* (EF_1 factors).
- **E3**: the “3D virtual reality model of the Church of Santa Maria Paganica in L’Aquila”, allows visitors to explore an important historic site destroyed by the earthquake of April 6th, 2009. By means of a game pad, users can approach artistic details, such as the Church choir, or move away out of the church to visit the square, or magically teletransport themselves onto the platforms built to observe the cupola artistic works. This scenario and associated activities contributed primarily to the evaluation of *interaction ability in using the game pad* (usability factor) and *exploration* of the virtual world (EF_1 factor).
- **E4**: in the “Blocks game” available on Leap Motion Store, users can interact with virtual blocks, moving them, creating them in different geometric forms and magically levitating them, by using their virtual hands, thanks to the Leap motion technology [114]. This scenario and associated activities contributed primarily to the evaluation of *interaction ability* via *gestures* (usability factor).
- **E5**: In the “Michelangelos’ David demo” available for the HoloLens Commercial Suite, users can interact with the photorealistic hologram of the statue using their own hands: they can approach the sculpture to discover the overall artistic details, miniaturize it, restore its original size, or even change it by chiseling the marble. This scenario and associated activities contributed primarily to the evaluation of *emotional participation in photorealistic images*, *body participation* and *action* (EF_1 factors), and *interaction ability* via *gestures* (usability factor) in a mixed reality setting.

### 2.5. Activities and Procedures

The study included two separate evaluation sessions with different settings and locations for the HoloLens and the Oculus Rift experiences: the *HoloLens session* took place in Milan at the Microsoft Lab with the support of two Microsoft people, while the *Oculus session* took place at TetaLab.

Each evaluation session was structured as a sequence of customary nurturing, body and closing phases [115,116]. During the *plenary nurturing phase*, operators introduced themselves to all participants, explained the objective and the overall organization of the experiment, and informed the participants that they could signal any discomfort at any time and that they could withdraw at any time by dismounting the headsets or by asking operators to dismount it. The *body phase* was organized as a sequence of sub-sessions, one for participant, each with a duration of approximately 20 min for the immersive experience followed by approximately 10 min for a short interview to obtain feedback from the participant. During the *plenary closing phase*, participants had a snack and share impressions about the experience in a friendly atmosphere while operators reordered collected material and annotated first impressions.

During individual sub-sessions, all participants were administered the activity listed in Table 4, requested with the same order to each participant (for the Oculus Rift session the order was: A1, A3, A4, A2, A1, A5, A6, A2; for the HoloLens session the order was: A1, A3, A4, A5, A6, A2). In the case of the Oculus Rift, participants were asked to dismount and re-mount the headset after the first two activities with the multifold objective of (1) ensuring a brief rest, (2) keeping them active during the preparation of the following scenario, and (3) having an additional test on the autonomy in handling the device.

The Concurrent Think Aloud (CTA) was used as a moderating technique to allow operators to understand participants’ thoughts while they interacted with the IVEs (CTA had no negative side effect on usability evaluation since accuracy and/or of time spent on a task were not to be measured) [116]. The controlled observational method [116] was used as data collection technique, given that all sessions took place in a laboratory setting. In order to obtain as much accurate data as possible, each sub-session was video-recorded by a camera; furthermore, during each individual sub-session, three operators took note of their observation (in summary, both direct and indirect behavioral observations were used). Operators included psychologists trained in evaluating ASD people behaviors and computer scientists with an HCI background and two years experience in TetaLab activities.

### 2.6. Ethical Considerations

Considerations about ethics and safety was carefully addressed, due also to the scarcity of related literature. Prior to the experiment, approval was obtained from the Ethics Committee of the researchers’ institution (prot. 19/2016). Study participants had been involved since more than one year in TetaLab activities, and this helped in the preparation of the experiment and in the exchange of necessary information and explanation. In order to ensure that we were not proceeding with an experience that might turn uncomfortable or anyway inappropriate for study participants, a preliminary evaluation session was conducted by four psychologists who individually examined demos, applications, each selected scenario and each selected activity, and evaluated them with respect to possible hazards in ASD people. During the actual experiment, participants were continually monitored.

## 3. Results

In the following, results on engagement—which is main objective of the study—are fully reported, while results on acceptability and usability are summarized (a complete report can be found in [117]). In order to provide a structured and easy to grasp summary presentation of results, Table 5, Table 6, Table 7, Table 8 and Table 9 present figures achieved in the Hololens session and figures achieved in the Oculus session paired by topic. Please notice that in no way is this to be intended as a comparison among the two HMDs aimed at determining which one is “better”, since the two devices provide intrinsically different experiences. The goal here is to highlight strengths and weaknesses of each device to later analyze how such strengths and weaknesses can be considered and addressed in the conception of HMD-based ASD treatment, as well as to reason on the results achieved in the two evaluation frameworks.

### 3.1. Acceptability and Usability

As to *acceptability*, all factors received a positive Boolean value: all the participants were willing to wear the HMDs and able to mount and dismount the headsets without support from operators and completed all the proposed scenarios; no participant reported and/or showed negative sensory or physiological experiences. All the participants were enthusiastic to participate to both sessions (this was is in particular remarkable for the HoloLens session, for which they chose to face a somehow burdensome trip to Milan while also overcoming physical impairments and/or social phobias).

As to *usability*, quantitative objective data were extracted from collected materials (notes and audio/video recording) and expressed according to a Low-Medium-High scale with Low = *v* ∈ [0–33%], Medium = *v* ∈ [34–66%], High = *v* ∈ [67–100%], where the meaning of *v* depends on the particular metrics of the evaluation framework. Specifically, for the *autonomy in managing the devices* group, we determined the number of times an ASD student asked for support with respect to the mean of this measure on Typical Development (TD) people and the time spent for mounting the HMD with respect to the mean of this measure on TD people (the control group included five graduated and undergraduate students from Psychology and Computer Science courses in the same age range of study participants); for metrics in the *comprehension* group, *v* is the number of recognized elements with respect to the total requested by the activity; for metrics in the *interaction ability* group, *v* is the number of correct actions with respect to the total requested by the activity. The Low, Medium, and High values were then mapped onto a classical *F* (Failure), *P* (Partial success) and *S* (Success) triad values (using an inverted philosophy in those cases where the fewer the better) and the success rate calculated as (*tot_S_* + 0, 5 ∗ *tot_P_*)/*tot_O_*, where *tot_S_* is the number of occurrences of *S*, *tot_P_* is the number of occurrences of *P*, and *tot_O_* is the total number of occurrences. Results are summarized in Table 5, which depicts overall mean scores along with standard deviation.

Possible fears on the cumbersomeness of HMDs seem to be overcome by our results both on acceptability and on the autonomy in managing the device. The partial success of *comprehension of VE features* (M = 0.53 and SD = 0.37 for HoloLens; M = 0.58 and SDS = 0.33 for Oculus Rift) is coherent with the need of initial training experienced also by typically developing people attending the lab; further investigation and studies on the medium or long term are needed to evaluate to what extent and with which learning rate practice may improve this scores; anyhow, the slight improvement from the first to the second session—despite the greater complexity of the Oculus interactive environments and the lower directness of the associated interaction modality—seems encouraging in this sense. The successful scores in *autonomy in managing the device* (particularly for the HoloLens) and in *interaction ability* open interesting opportunities and research lines for innovative interventions, as we will discuss further on.

### 3.2. Engagement

Quantitative and qualitative data relating to behavioral observation were extracted from collected materials (notes and audio/video recording), according to the metrics/activity association defined in Table 6 (notice that the evaluation of emotional participation in watching non-photorealistic images was feasible only in the Oculus Rift session).

*Factors in EF_I* have been evaluated according to a customary [1,2,3,4,5] Likert scale where 1 = very poor, 2 = poor, 3 = average, 4 = good, and 5 = very good. For each participant in each session, three observers individually assigned a score for each factor; then, for each factor of each participant in each session the average score was calculated. Table 7 depicts such scores, along with the overall mean scores and standard deviation for each factor in each HMD; the mean normalized values are then computed to facilitate the comparison with factors in EF_II.

Notable quantitative findings are related to *suspension of disbelief*, below average for the HoloLens (0.58) and slightly above average for the Oculus (0.64), confirmed also by qualitative findings during the sessions, with participants reporting the perception of the distinction between the real and the virtual world. Consolidated studies on immersive environments would suggest it be regarded as a not so positive result (the lower the suspension of disbelief, the lower the degree of presence achieved is expected to be and, consequently, the lower the efficacy of the immersion is expected to be). The average score of *body participation* (i.e., the motor response to IVE events) suggests that practice is needed to feel free to physically move during an immersive experience. We recall that in this case we evaluated the degree of coherent body reactions to what they were experiencing within the virtual environment (e.g., if they moved their arms when in the environment they were flying). The difference between the scores in HoloLens (0.68) and Oculus (0.44) suggests that the cable connecting the Oculus Rift headset to the computer may have had an impeding role; we expect that better results would be achieved with the more recent cordless version of the Oculus (additional experiments are necessary to confirm such a hypothesis). The average score of *exploration* (i.e., the degree of voluntary exploration of the virtual synthetic world) suggests that practice is needed also to feel free to virtually move. The lower score of the HoloLens (0.65) might be due to the fact that the HoloLens session took place in an unfamiliar setting with two unfamiliar persons from Microsoft giving support, which, for ASD people, might be intimidating (his hypothesis seems confirmed also by results in EF_II, as discussed later on). Additional experiments would be appropriate also to evaluate a possible interdependency between body participation and IVE voluntary exploration: to what extent the movement constraints in the physical world affected the sense of agency and control in the virtual world? (according to [100], “I am present in a real or virtual space if I manage to put my intentions into action (enacting them)”).

*Factors in EF_II* have been evaluated according to the qualitative tags defined in Table 2 in post-experiment sessions, in which the observers reviewed all collected materials (notes and audio/video recording). Table 8 depicts the tags assigned to each factor for each participant in each session. Then, in order to have a numerical reference and facilitate the comparison with factors in EF_I, as for usability metrics, the scores were then mapped onto a *F* (Failure), *P* (Partial success) and *S* (Success) triad values and the success rate calculated as (*tot_S_* + 0, 5 ∗ *tot_P_*)/*tot_O_*, where *tot_S_* is the number of occurrences of *S*, *tot_P_* is the number of occurrences of *P*, and *tot_O_* is the total number of occurrences. These overall mean scores along with standard deviation are depicted in Table 8 for each factor and each HMD. Results show a somewhat homogenous behavior with the exception of *verbal reaction* in the HoloLens session; again, this might be explained by the somewhat intimidating interaction with unfamiliar people from Microsoft. In addition, in the case of *facial expression*, the HoloLens score (0.7) is lower than in the Oculus session (0.9), while *level of attention* and *emotional participation* are not so different. As to *facial expression*, it has also to be observed that changes in the expression may be minimal in ASD persons: several studies demonstrate reduced facial expressiveness, or “flat affect” in ASD, compared to spontaneous expressiveness that occurs during natural interaction [118]. Hence, even the minimal changes revealed by the behavioral observation during the experiment are to be considered positive and a signal of engagement.

Overall, according to EF_II, the engagement is to be judged high for both HMDs. This result appears to be in slight contrast with figures related to EF_I, that, for both HMDs, seem to rather suggest a medium degree of engagement (see Table 9 for a summary comparison).

This disagreement between the overall scores of two sets of engagement factors, consistent in both HMDs, provides a positive answer to RQ1 (“*can the immersive experiences be considered actually engaging (and hence promote the HMDs as potentially appropriate for treatment support) even when IVE-specific evaluation metrics seem to suggest a not optimal level of engagement?*”) and stimulates reflections about the interpretation we must give to the scores of EF_I metrics in case of ASD people. Is the higher still the better? Or, as asked by RQ2: *can the lack of success in any IVE-specific evaluation metrics turn out to be potentially useful within the framework of IVE-based ASD treatment design?* We analyze this issue in the Discussion.

## 4. Discussion

In this study, we evaluated the appropriateness and engagement of two HMDs (Oculus and Hololens) in a group of five young adults with high-functioning ASD, as well as the efficacy of the selected metrics for the measurement of the engagement. In a broader perspective, the aim of our study is to provide indications on the potential use of IVE-based treatment in ASD. In recent years, there has been a growing interest in the use of VR/MR technologies in clinical and educational contexts for autism, demonstrating how the use of ICT-based devices are more engaging, attractive and motivating for people with ASD [56,119,120]. Different from other studies on the use of immersive virtual environments in the treatment of ASC based on self-reports, this study relies on behavioral observation to avoid potential errors and biases due to the difficulty of people with ASD to reflect and report on their behavior and emotions.

### 4.1. Acceptability and Usability

Our results support the accessibility of both devices experimented by all participants. These findings are in line with recent literature suggesting that VR tools are widely accepted and appreciated among individuals with ASD [63,121,122]. Furthermore, it is interesting to note that none of our participants reported adverse effects such as discomfort, cybersickness or sensory problems, and all participants completed all scenarios. As suggested by [121], the issue of acceptability is of fundamental importance for the use of VR devices in clinical and intervention settings. The lack of acceptability of an intervention method can actually influence the course and outcome of a treatment [123]. Regarding the usability dimension, in terms of *autonomy in device management*, *understanding of IVE characteristics* and *interaction abilities*, we note differences between the two devices. Specifically, the HoloLens session seems to have favored greater autonomy in device handling than the Oculus session. On the other hand, the interaction ability seems to be better during the Oculus session. In any case, an initial training session seems to be necessary to ensure greater autonomy, as demonstrated by the partial success in understanding the features and functionality of IVEs.

### 4.2. Engagement

Interesting results come from the analysis of engagement factors. Since according to EF_II the engagement is evaluated as definitely high, one would expect to detect high values also in the IVE specific evaluation factors, for which, in TD people, “the higher the better”. Conversely, values achieved in both HMDs show a clear discrepancy, reaching a medium level. *Emotional participation in images* and *suspension of disbelief* are maybe the most interesting results, somewhat deviating from what is expected in TD people, but for this reason providing useful suggestions, as to content issues, nature of applications, and ASD-specific interventions, as discussed in the following.

#### 4.2.1. Degree of Realism

Since *photorealism* is generally suggested to play a critical role in the achievement of engagement (due to the higher fidelity of the IVE with respect to the real world), the high flexibility of the two investigated HMDs and their high degree of “reproduction fidelity” (defined by [58] as the relative quality with which the synthesizing display is able to reproduce the actual or intended images of the objects being displayed) would in principle direct designers towards choices based on a high degree of realism for 3D scenarios to be used in treatment procedures.

Conversely, our results on the emotional participation in photorealistic and non-photorealistic images seem to suggest a design direction based on the integration of multimedia information at different level of realism (e.g., video, photographs, and cartoon-like images and animations). Indeed, we achieved almost the same score for emotional participation in photorealistic and non-photorealistic scenarios in the Oculus session (which offered both cases). This result may be due to the fact that ASD people become easily distracted by irrelevant details of a scene and feel more comfortable in facing simplified reality and/or to the fact that, due to their perceptual difficulties, they do not perceive the scene in the same way as TD people, as observed also by [55]. The combination of these factors makes the realistic nature of 3D scenes less important for ASD people than TD people, confirmed also from individual interviews with participants conducted after the immersive experience, where it clearly emerged that a factor more important than photorealism for “emotional participation in images” was the subjective familiarity with IVE objects, which may explain the lower score for emotional participation during the HoloLens session.

It has been suggested that the main feature of electronic screen media which renders them ideal for the delivery of information to ASD people is the relatively constrained screen viewing area limiting the attentional frame and helping those with ASD to focus their attention on relevant stimuli while ignoring irrelevant ones [25,124,125,126]. Moving from a clearly delimited screen to the surrounding effect of an immersive experience might be detrimental in this sense. Anyhow, our results on the appreciation of non-photorealistic images suggest that stimuli reduction might be achieved by acting upon the photorealism degree and the scene complexity. In particular, from a rehabilitation perspective, we believe that it could be useful to build environments that are able to progress “gradually” from less realistic scenarios towards environments that are more representative of the real-world. Specifically, rehabilitation interventions should be based on virtual environments with varying degrees of realism and immersiveness, personalizable, and with progressive adaptation. This should facilitate the transfer of skills learned in the virtual environment into everyday life. Furthermore, this process could also be supported by auditory soundtracks closely synchronized with the visual stimulation, so that the multimodal information flow of the immersive experience assists the user in the coordinated processing of information [25,27].

#### 4.2.2. Suspension of Disbelief

Regarding the suspension of disbelief, generally considered a key aspect in interacting with IVEs [53], the participants in our study showed a continuous and consistent awareness of the distinction between the real and virtual worlds. For example, some participant stated that “one cannot be frightened by a fictitious world”. This difficulty in achieving a complete suspension of disbelief is not necessarily negative but, on the contrary, can be a strength for the structuring of rehabilitation interventions based, for example, on systematic desensitization and gradual exposure within non intimidating virtual environments. This technique is widely used for the treatment of phobias and anxiety disorders and, in the last decade, has also begun to be employed with the aid of VR in people with autism [42,64]. The most recent literature demonstrates that people diagnosed with ASD are characterized by high levels of anxiety that have a significant impact on daily life and quality of life [127,128]. Furthermore, individuals with ASD may develop unusual phobias or social anxiety that is not related to embarrassment or fear of “negative judgement” but rather to “social confusion” resulting from their difficulties in processing and understanding information and social contexts [129,130]. In addition, the presence of sensory anomalies and anxiety often cause avoidance of certain contexts and activities (e.g., public transport, supermarkets, bars, hospitals, restaurants, etc.), significantly limiting independence and autonomy even in adulthood.

Virtual reality environments can be a powerful training tool, as they provide safe and controlled environments in which scenarios that the person in real-life experiences as anxiety-provoking are proposed. One of the fundamental advantages of using of VR in treatments is that of being able to include objects and/or situations that are difficult to find/use in the real environment and to control the complexity of the required social interaction so to make the simulation gradually evolve towards its real-life counterpart. For example, researchers [34,36,42,131] have designed VEs for understanding social scenarios (bar, bus, job interview, parties, meeting with friends or strangers) or for promoting adaptive skills (road safety, driving simulations, supermarket, airport). This would favor the desensitization process towards situations experienced as potentially stressful, anticipating their contents.

Augmented reality as well may be beneficial as to anxiety reduction: we believe that the low scores obtained during the Hololens session with regard to the suspension of disbelief, combined with more autonomy in managing the device and good score in the exploration and action aspects is an advantage for its potential use as a prosthetic tool capable of enriching the real world with familiar objects and making it less scary

#### 4.2.3. Analysis of Factors in EF_II

In our study, we also considered more qualitative involvement factors (EF_II) that can be interpreted as indicators of both cognitive and emotional involvement. Our results show that the activities proposed in the IVEs are able to capture the participants’ interest in the specific activity, especially during the Oculus session, showing positive scores in terms of facial expressiveness, focused attention, emotional participation, and verbal reactions. It is worth emphasizing that the factors analyzed represent aspects that are often impaired in autism compared to individuals with typical development; therefore, the results obtained provide further confirmation of the effective engagement capacity of immersive experiences in ASD. A prerequisite for effective learning is to capture attention and motivate the individual to continue the activity. During the experiments, our participants demonstrated active engagement, indicated by verbal reactions with frequent questions about the frameworks and technical specifications of the activities, both during and after the sessions. This result also suggests that the proposed activities aroused a certain degree of curiosity associated with cognitive engagement, with good levels of focused attention to processing and understanding of the proposed activities. Promising results also seem to be those related to emotional engagement, with positive affective attitudes before and during the experimental sessions suggesting pleasure, enthusiasm and excitement for the activities. This may be regarded a surprising result considering that ASD persons may present resistance to change or to facing new situations [132].

#### 4.2.4. Limitations

While results of this study reveal interesting findings and suggest promising research lines, some limitations are to be underlined (also observed in other similar studies). The first one is related to the length of the study and the consequent limited amount of exposure to HMDs and IVEs that the participants received; additional study is necessary to evaluate whether a longer exposure might yield different results. Another limitation is due to the possible bias deriving from the fact that all participants had been previously involved in TetaLab activities (though not related to immersive experiences); additional experiments with different high-functioning users in the same age range would be appropriate for a better generalization of results. Finally, an obvious limitation is given by the small size of the user sample; however, as observed in [60], even if most studies are conducted with small user groups, this limit can be outbalanced by the numbers of studies collectively contributing. To the best of our knowledge, our study provides the first contribution specifically focused on young high-functioning adults, a user group with great potential with respect to both the willingness to adopt VR technology and the inclusion in social and working life. Therefore, we encourage other researchers in the field to carry out additional studies in this direction on this specific users’ population. We also underline that the homogeneity of the users’ sample makes results more generalizable than in studies with heterogenous samples.

## 5. Conclusions

In this paper, we presented the results of a study aimed at evaluating the engagement capability and the potential appropriateness of two HMDs with respect to technology-enhanced ASD treatment, as well as at investigating the indication provided by engagement evaluation metrics and their translation into guidelines for VR/AR-based ASD treatment design. We tried to pave the way towards the definition of methods and techniques overcoming recognized limitations of existing proposals as to assessment aspects in the ASD population: evaluation is typically based on self-reporting, no real consensus on which metrics to use does exist, generally adopted metrics are the ones conceived for typical development people, study participants are generally quite heterogenous, and most of the study are focused in children, leaving other ASD people under considered. All these weaknesses make the evaluation of VR/AR-based ASD treatment still an open problem. Differently from other studies, our study contributes to such open problem by: (1) considering a homogeneous users’ sample, (2) specifically focusing on a specific users’ population under considered in the literature while potentially highly open to the utilization of VR/AR technology, (3) conducting the evaluation according to behavioral observation, (4) reasoning on the contribution that metrics of a different nature may give to the evaluation in the case of ASD population, (5) reasoning on how partial failures of some metrics may be actually positively taken into account for the definition of the treatment. In particular, as to the last two points, engagement was investigated according to two sets of metrics (respectively focused on (1) factors specifically associated with the evaluation of IVE and (2) factors typically associated with the evaluation of engagement in generic situations, in order to determine whether—in the case of ASD people—the immersive experience can be anyhow engaging even when some IVE-specific evaluation metrics seem to suggest differently (RQ1) and whether the lack of success of some IVE-specific evaluation factor can actually become an advantage from the perspective of a treatment (RQ2). Throughout the paper, the working hypothesis, findings, and implications have been discussed, taking always into account related studies (actually, the paper provides an extensive review of the literature).

The study provided positive answers to both research questions and our results suggest that the use of IVEs in ASD intervention settings is a promising approach that can increase the effectiveness of learning processes, eliminate environmental distractions, and promote the maintenance of concentration, in part through the structuring of predictable and simple environments experienced as less stressful, thus confirming the results of previous studies [60,121]. In particular, as to possible applications in ASD interventions, our results suggest complementary roles of the two headsets, with totally immersive VR (e.g., experienced by Oculus Rift) more appropriate for learning applications and augmented reality (e.g., experienced by HoloLens or smart glasses) utilized as a prosthetic tool able to enrich the real world with objects familiar to the ASD persons, helping them to cope anxiety, fear and phobias in social situations.

We are currently working on these two complementary directions within the framework of a project financed with public funds. VR/AR-based interventions and treatment are being defined as bridges to the real world, according to design choices taking into account ASD people’s strong visual memory [60], visuospatial modalities, predictability and repeatability provided by IVEs, to capture the interest of the ASD person and increase their attention and motivation toward social stimuli in the environment [133], by gradually shifting the focus of attention from non-social, inanimate objects to more complex social stimuli and situations.

As a final consideration, we observe that ASD impacts an individual’s development and adaptation across their lifespan, and the typical symptomatology of the condition causes significant distress in daily life, even in the presence of adequate cognitive functioning. VR/AR-based interventions can have a strong social and economic impact by improving activities necessary for independent living, such as communication and social skills, as well as community and adaptive skills (e.g., driving, taking public transport) and employability. Complete autonomy and integration into the community often remain a challenge even for people with high-functioning ASD (such as in the case of the users’ population considered in our study); providing effective solutions can really improve the quality of life for people with autism and their families.

## Figures and Tables

**Table 1:** Characterization and IQ of study participants.

	P1	P2	P3	P4	P5
**Age**	21	21	23	21	22
**Gender**	M	M	M	M	M
**Years of education**	16	16	16	15	13
**Social cognition measures**					
**CE-BES**	30	37	34	30	25
**AE-BES**	43	45	39	44	38
**EYES TASK**	19	25	18	18	23
**Advanced ToM**	11	12	12	10	12
**Wechsler Adult Int. Scale (WAIS)**					
**WAIS-VCI**	110	96	69	86	98
**WAIS-PRI**	94	114	73	73	83
**WAIS-WMI**	89	89	83	92	92
**WAIS-PSI**	83	89	97	75	75
**WAIS-IQ**	94	98	73	76	84

**Table 2:** The adopted engagement evaluation framework.

**ENGAGEMENT FACTORS I (EF_I)—5-point Likert Scale**			
**Emotional part. in images I (Ph)**	participation in watching photorealistic images		
**Emotional part. in images II (NPh)**	participation in watching non-photorealistic images		
**Suspension of disbelief**	the extent to which the virtual world is temporarily accepted as reality		
**Body Participation**	the extent of body movement during the IVE experience, measured as the degree of coherent body reactions with respect to expected and allowed body movement		
**Exploration**	the degree of voluntary exploration of the virtual synthetic world		
**Action**	the degree of voluntary participant’s input actions not mandatorily required from the activity		
ENGAGEMENT FACTORS II (EF_II) *—qualitative evaluation			
**Facial expression**	negative	neutral	positive
**Level of attention**	fleeting	vigilant	focusing
**Emotional participation (overall)**	low	medium	high
**Verbal reaction**	not present	shallow	deep

* Evaluated throughout the entire experience.

**Table 3:** Association between IVEs and the engagement evaluation framework.

EF_I Factors	IVEs
Emotional part. in images I (Ph)	E1, E2, E3, E5
Emotional part. in images II (NPh)	E2
Suspension of disbelief	E1, E2, E3, E5
Body Participation	E1, E2, E3, E5
Exploration	E1, E3, E5
Action	E3, E5
**EF_II factors**	All IVEs for each factor

**Table 4:** Performed activities and associated interaction modality. One may notice that the only interaction modality with the environment experienced via the HoloLens was by *gestures*, while in the case of the Oculus Rift session, available interaction modalities include also *remote control* and *game pad*.

Activities	Description *	Interaction Modality	
		*HoloLens*	*Oculus Rift*
A1	Mounting the HMD	-	-
A2	Dismounting the HMD	-	-
A3	Browsing menus	gesture	remote control
A4	Watching IVE	-	-
A5	Exploring IVE	gesture	game pad
A6	Playing with IVE	gestures	gestures

* Specifics of activities A3–A6 in different IVEs were tuned to the specifics of the IVE.

**Table 5:** Summary of usability evaluation: mean scores with standard deviation.

Metrics	HL Activities	OR Activities
Autonomy in managing devices	0.90 (0.20)	0.75 (0.25)
Comprehension of IVE features	0.53 (0.37)	0.58 (0.33)
Interaction ability	0.70 (0.24)	0.77 (0.31)

**Table 6:** Association between metrics and HoloLens (HL)/Oculus Rift (OR) activities.

**ENGAGEMENT FACTORS I**		
**EF_ I Metrics**	**HL Activities**	**OR Activities**
Emotional part. in images I (Ph)	A4	A4
Emotional part. in images II (NPh)	-	A4
Suspension of disbelief	A3, A4, A5, A6	A3, A4, A5, A6
Body Participation	A3, A4, A5, A6	A3, A4, A5, A6
Exploration	A3, A4, A5, A6	A3, A4, A5, A6
Action	A3, A4, A5, A6	A3, A4, A5; A6
**ENGAGEMENT FACTORS II**		
**EF_ II Factor**	**HL Activities**	**OR Activities**
Facial expression	A1, A2, A3, A4, A5, A6	A1, A2, A3, A4, A5, A6
Level of attention	A3, A4, A5, A6	A3, A4, A5, A6
Emotional participation (overall)	A3, A4, A5, A6	A3, A4, A5, A6
Verbal reaction	A1, A2, A3, A4, A5, A6	A1, A2, A3, A4, A5, A6

**Table 7:** Results for engagement factors I: mean (M) scores with standard deviation (SD) and normalized (NORM) mean scores.

**ENGAGEMENT FACTORS I—HoloLens**						
**ID**	**Emotional Participation in Images**		**Suspension of** **Disbelief**	**Body** **Participation**	**Exploration**	**Action**
	**Ph**	**NPh**				
**P1**	4.00	-	3.00	3.00	3.00	4.00
**P2**	4.50	-	3.00	2.00	2.50	4.00
**P3**	3.00	-	2.00	4.00	3.00	3.50
**P4**	3.25	-	3.00	4.00	4.00	3.00
**P5**	5.00	-	3.50	4.00	3.75	5.00
**M (SD)**	3.95 (0.75)	-	2.90 (0.49)	3.40 (0.8)	3.25 (0.55)	3.90 (0.66)
**NORM**	0.79	-	0.58	0.68	0.65	0.78
**ENGAGEMENT FACTORS I—Oculus Rift**						
**P1**	3	3.66	3	1.66	3.33	4.5
**P2**	4	4.66	3	2	3.66	3.66
**P3**	4.66	4	3.33	2.66	3.66	3.66
**P4**	4.66	3.66	3.33	2.66	2.66	2
**P5**	4.66	4	4.33	2	3.66	4.66
**M (SD)**	4.20 (0.65)	4.0 (0.37)	3.40 (0.49)	2.20 (0.40)	3.39 (0.39)	3.70 (0.94)
**NORM**	0.84	0.8	0.64	0.44	0.70	0.74

**Table 8:** Results for engagement factors II: mean (M) scores with standard deviation (SD).

**ENGAGEMENT FACTORS II (EF_II)—HoloLens**				
**ID**	**Facial Expression**	**Level of Attention**	**Emotional Participation (Overall)**	**Verbal Reaction**
**P1**	neutral	focusing	medium	not present
**P2**	positive	focusing	high	shallow
**P3**	neutral	focusing	medium	shallow
**P4**	neutral	focusing	medium	deep
**P5**	positive	focusing	high	deep
**M (SD)**	0.7 (0.24)	1 (0.00)	0.7 (0.24)	0.6 (0.37)
**ENGAGEMENT FACTORS II (EF_II)—Oculus Rift**				
**P1**	neutral	vigilant	medium	deep
**P3**	positive	focusing	high	deep
**P4**	positive	focusing	high	deep
**P5**	positive	focusing	medium	shallow
**P6**	positive	focusing	high	deep
**M (SD)**	0.9 (0.20)	0.9 (0.20)	0.8 (0.24)	0.9 (0.2)

**Table 9:** Engagement factors—Summary results.

**ENGAGEMENT FACTORS I—Summary Results**						
	**Emotional Participation in Images**		**Suspension of** **Disbelief**	**Body** **Participation**	**Exploration**	**Action**
	**Ph**	**NPh**				
**HL**	0.79	-	0.58	0.68	0.65	0.78
**OR**	0.84	0.8	0.64	0.44	0.70	0.74
**ENGAGEMENT FACTORS II—Summary results**						
	**Facial Expression**	**Level of Attention**		**Emotional Participation (overall)**		**Verbal Reaction**
**HL**	0.7	1		0.7		0.6
**OR**	0.9	0.9		0.8		0.9

## Data Availability

The data presented in this study are available in [117].
